# Supplementary material for: The Consortium for Genomic Diversity, Ancestry, and Health in Colombia (CÓDIGO): building local capacity in genomics and bioinformatics
Source: Commun Biol. 2025 Jul 17;8:1062. doi: 10.1038/s42003-025-08496-9 (PMC12271396; doi:10.1038/s42003-025-08496-9)

The Consortium for Genomic Diversity, Ancestry, and Health in Colombia (CÓDIGO): building local capacity in genomics and bioinformatics

Leonardo Mariño-Ramírez, Shivam Sharma, James Matthew Hamilton, Thanh Long Nguyen, Sonali Gupta, Aravinth Venkatesh Natarajan, Shashwat Deepali Nagar, Jay Landon Menuey, Wei-An Chen, Adalberto Sánchez-Gómez, José María Satizábal-Soto, Beatriz Martínez, Javier Marrugo, Miguel A. Medina-Rivas, Juan Esteban Gallo, I. King Jordan, Augusto Valderrama-Aguirre

Supplement Contents

Supplementary Note. **Sample provenance and ethics approval.** S2

Supplementary Figure 1. **Variant merging and harmonization** S3

Supplementary Table 1. **CÓDIGO datasets** S4

Supplementary Table 2. **Global reference populations** S4

Supplementary Figure 2. **CÓDIGO development stack** S5

Supplementary Figure 3. **Overlap of variant sites among CÓDIGO datasets.** S6

Supplementary Figure 4. **K-means clustering elbow plot** S6

Supplementary Note. **Sample provenance and ethics approval.**

All study participants provided written informed consent for the use of their DNA in genetic studies. Study participant samples and Institutional Review Board (IRB) approval was obtained for each CÓDIGO dataset as detailed below.

Afro-Colombian from Chocó (CHG): Samples and ethnical approval from The ChocoGen project of the Universidad Tecnológica del Chocó (PMID: 27668076, 28855283).

Afro-Colombian from San Basilio de Palenque (PLQ): Samples and ethnical approval from The Proyecto Genes Candidatos en Asma (PGCA) study of the Universidad de Cartagena (PMID: 27725671).

Indigenous Arhuaco, Curripaco, Emberá, Guahibo, Inga, Kogi, Piapoco, Waunana, Wayuu communities (IND): Ethical approval for the sample characterization was obtained from the National Health Service National Research Ethics Service, Central London PMID: 22801491) with Colombian samples provided according to the terms of a data use agreement from the Universidad de Antioquia and with IRB approval from the Georgia Institute of Technology (PMID: 31545791).

Indigenous Sinú community (SIN): Samples from the Population Architecture using Genomics and Epidemiology (PAGE) consortium with ethical approval from the Universidad de Cartagena (PMID: 31217584).

Mestizo Colombian from Medellín (CLM): IRB approval was obtained as part of the 1000 Genomes Project from the Coriell Institute, which maintains the CLM cell line (PMID: 26432245), and the University of Antioquia, where the samples were collected (PMID: 18369456).

Mestizo Colombian from Medellín (MCM): Approval was obtained from the ethics committee and the research committee of Universidad CES (PMID: 30967898).

Mestizo Colombian from Medellín (MCA): Samples from the Consortium on the Genetics of Schizophrenia (COGS) (PMID: 31591465) with approval from the ethics committee and the research committee of Universidad CES (PMID: 30967898).

Mestizo Colombian from Valle del Cauca (VDC): Samples from the Genomic Variability of the Mucopolysaccharidosis Complex in Southwestern Colombia project and ethical approval obtained from the School of Health, Universidad del Valle.

Supplementary Figure 1. **Variant merging and harmonization.**

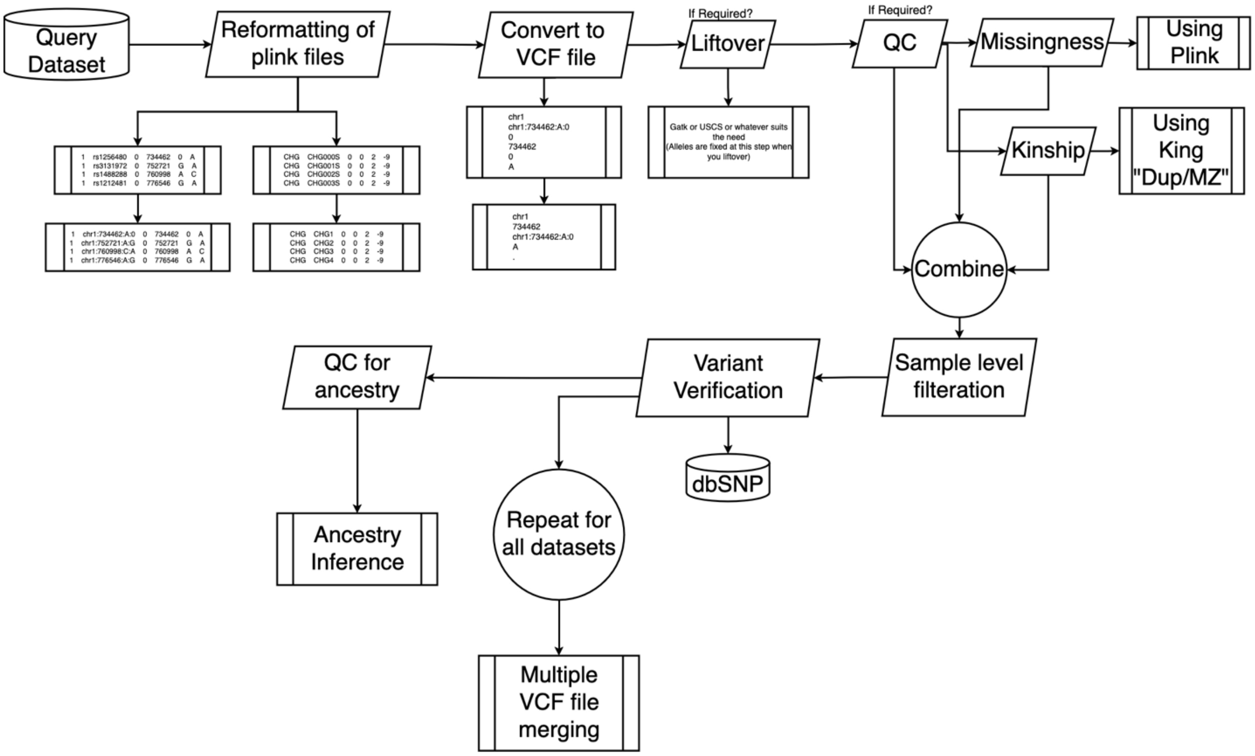

Supplementary Table 1. **CÓDIGO datasets.** The numbers (n) of samples and genomic variants before and after variant merging and harmonization are shown for each source dataset.

Source code[a]	Source description[b]	n samples before	n variants before	n samples after	n variants after
CHG	Afro-Colombian from Chocó	100	568,662	100	567,184
PLQ	Afro-Colombian from San Basilio de Palenque	34	10,064,050	34	9,779,781
IND	Indigenous Arhuaco, Curripaco, Emberá, Guahibo, Inga, Kogi, Piapoco, Waunana, Wayuu communities	50	364,430	50	296,141
SIN	Indigenous Sinú community	19	1,407,123	19	779,200
CLM	Mestizo Colombian from Medellín	94	81,568,731	94	81,568,727
MCM	Mestizo Colombian from Medellín	524	24,181,263	373	23,188,190
MCA	Mestizo Colombian from Antioquia	624	541,134	623	526,935
VDC	Mestizo Colombian from Valle del Cauca	116	6,481,171	116	6,481,171

[a] Three letter code for each source dataset
[b] Ethnicity and geographic origins for each source data set

Supplementary Table 2. **Global reference populations.** African, Admixed American, Indigenous American, and European reference populations and samples used for genetic ancestry inference. Population descriptions taken from The International Genome Sample Resource (ISGR): https://www.internationalgenome.org/data-portal/population. 1KGP = 1000 Genomes Project; HGDP = Human Genome Diversity Project.

Name	Description	Superpopulation	n	Source
Esan	Esan in Nigeria	African	99	1KGP
Gambian Mandinka	Gambian in Western Division, The Gambia - Mandinka	African	106	1KGP
Yoruba	Yoruba in Ibadan, Nigeria	African	107	1KGP
Karitiana	Karitiana in Brazil	Indigenous American	12	HGDP
Maya	Maya in Mexico	Indigenous American	13	HGDP
Peruvian	Peruvian in Lima, Peru	Admixed American	14	1KGP
Pima	Pima in Mexico	Indigenous American	12	HGDP
Surui	Surui in Brazil	Indigenous American	8	HGDP
British	British in England and Scotland	European	87	1KGP
Iberian	Iberian populations in Spain	European	86	1KGP
Toscani	Toscani in Italy	European	100	1KGP

Supplementary Figure 2. **CÓDIGO development stack.** Server-side components used for the CÓDIGO webserver are shown.

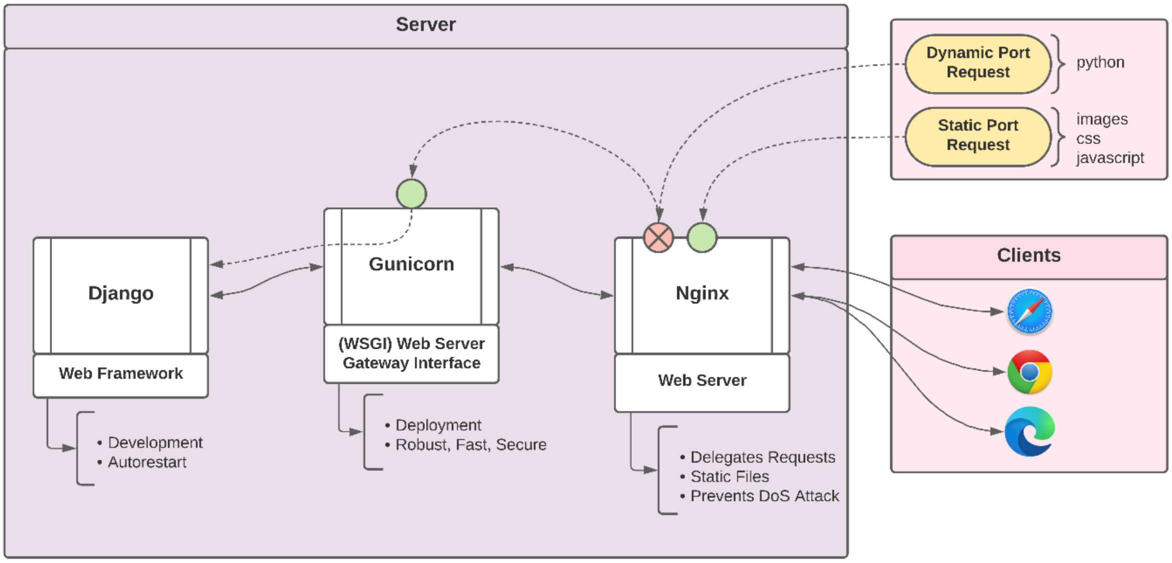

Supplementary Figure 3. **Overlap of variant sites among CÓDIGO datasets.** Upset plot showing numbers of variants for each CÓDIGO dataset and the extent of overlap among datasets. The top 30 overlaps are shown in the main plot, and inset shows the overlap among the WGG datasets. The genomic technologies used to characterize each dataset are color-coded as shown in the key: whole genome genotyping (WGG; yellow), whole exome sequencing (WES; blue), and whole genome sequencing (WGS; red).

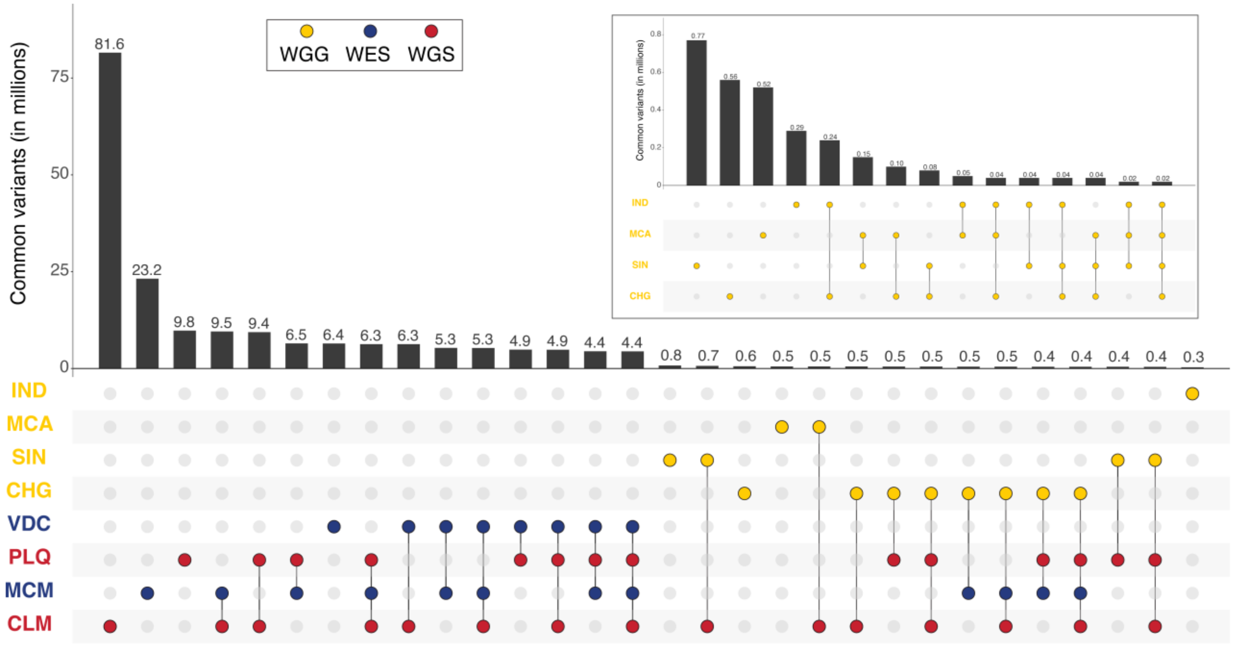

Supplementary Figure 4. **K-means clustering elbow plot.** Sum of squared errors (y-axis) plotted against the number of clusters (k; x-axis) for K-means clustering of CÓDIGO sample genetic ancestry fractions. K=5 yields the optimal number of genetic ancestry clusters for the CÓDIGO samples.

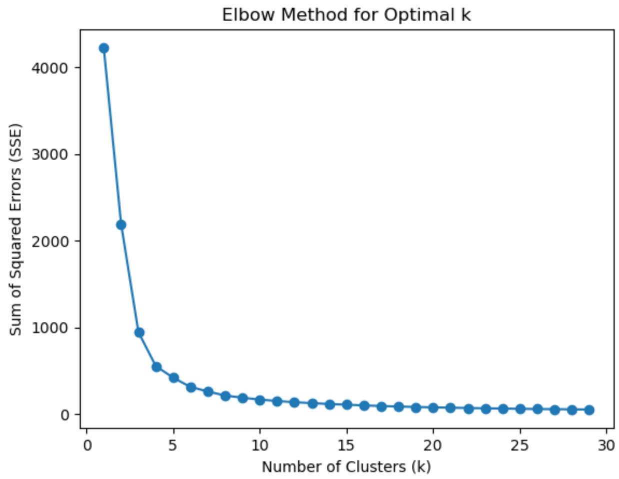

Supplement: Supplementary file 2 — Supplementary Information [file 42003_2025_8496_MOESM2_ESM.pdf]
