# Supplementary material for: The Consortium for Genomic Diversity, Ancestry, and Health in Colombia (CÓDIGO): building local capacity in genomics and bioinformatics
Source: Commun Biol. 2025 Jul 17;8:1062. doi: 10.1038/s42003-025-08496-9 (PMC12271396; doi:10.1038/s42003-025-08496-9)
Supplement: Supplementary file 3 — Description of Additional Supplementary Files [file 42003_2025_8496_MOESM3_ESM.pdf]

Description of Additional Supplementary Files

File name: Supplementary Data 1-2
Description: Ancestry-enriched variants with pharmacogenomic and clinical genetic annotations.
